# Supplementary material for: Are carbon emissions trading and green financial instruments synergistic? -Comprehensive quantitative research based on content analysis
Source: PLoS One. 2024 Mar 7;19(3):e0298601. doi: 10.1371/journal.pone.0298601 (PMC10919601; doi:10.1371/journal.pone.0298601)
Supplement: S1 Data — (ZIP) [file pone.0298601.s001.zip › Data/地级市Z_F工作报告.zip!/║┌┴·╜¡/╝╤─╛╦╣╩╨/└┤╫╘╣1⁄2╓┌║┼í╛┬φ┐╦╩2╛▌═°í┐_2011.docx]

各位代表：

现在，我代表市人民政府向大会报告工作，请予审议，并请各位政协委员和列席会议的同志提出意见。

一、2010年工作回顾

过去的一年，在市委的坚强领导下，我们紧紧依靠全市人民，坚定地贯彻落实科学发展观，奋力推进“两区两带一城”和十项重点工程建设，经济社会保持了平稳快速发展，圆满地完成“十一五”的各项目标任务。全市地区生产总值预计完成520亿元，同比（下同）增长18%。财政一般预算收入预计完成20.85亿元，增长43.4%，是“十五”末的3.86倍，年均增长30.8%。

——项目建设取得显著成效。全社会固定资产投资预计完成260亿元，增长50%。开工建设千万元以上项目345个，比上年增加71个，完成投资202亿元，增长45 .4%。其中产业项目开工建设140个，完成投资105亿元。北方公司日产5000吨水泥熟料生产线改造、中唯实业20万吨铸造焊接加工中心、葵花药业异地扩建、常发佳联农机等一大批新引进亿元以上项目相继开工建设。其中佳星公司日熔600吨超白玻璃和七彩毯业年产1000万条毛毯项目实现当年开工建设、当年竣工投产。农副产品加工、装备制造、能源产业实力大幅提升，风电装机突破80万千瓦，居全省领先地位。一批重大项目前期取得实质性突破，黑瞎子岛保护与开放开发上升到国家战略。谋划大型产业项目400项，总投资超过5000亿元，为项目可持续发展奠定了基础。一区多园建设全面推进，高新区获得省国土资源厅批复建设面积41.37平方公里，一期1.71平方公里，完成基础建设投资2.4亿元,开工建设项目16个，4个项目已建成投产。招商引资取得新进展，到位资金预计完成145亿元，增长57%；实际利用外资预计完成1.13亿美元，增长120.3%。

——工业经济实现三年翻番。预计全年规模以上工业增加值实现66亿元，销售收入实现300亿元，利税实现25亿元，分别增长40.7%、45.6%、47.9%，比2007年增长115%、144%、163%，三项经济指标均超额完成了三年翻番目标。优势产业支撑作用进一步增强，预计农副产品加工、装备制造、能源化工三大产业销售收入分别实现112亿元、72亿元和52亿元，总额占全市工业销售收入比重达到78.7%。骨干企业发展步伐进一步加快，预计全年销售收入超亿元的企业达到60户。其中超10亿元企业4户，超20亿元企业1户。产业项目建设和新产品开发快速推进。全市工业产业项目固定资产投资创历史最高水平，全年完成投资60亿元，三年累计完成投资160亿元，全年有72个产业项目投产。加强企业技术中心建设，完善自主创新体系，提高新产品研发和创新能力，全年有35项新产品投放市场。县域工业加快发展。全年县域工业预计实现销售收入125亿元，比上年增长54.7%。18户国有企业改制和166户企业改制遗留问题全部完成，8万名职工得到妥善安置，县（市）区81户企业改制全部完成。全市并轨企业拖欠职工工资全部解决。

——现代农业全面推进。坚持抗灾夺丰收，全市粮食产量达到110亿斤，创历史新高。建设了10万亩以上的优势种植产业带10条，规模化生产面积300万亩，实现了三年1000万亩目标。新组建投资千万元的农机合作社12个，农机总动力达到286万千瓦，农田综合机械化程度达到86.6%，保持全省领先。全市农田水利投入近3亿元，水利基础设施逐步完善，灌溉能力进一步增强。三大新建灌区和老灌区续建提升灌溉能力303万亩，水稻面积新增90万亩，达到448万亩。全市农业科技园区达到851个，带动重点农业技术覆盖率达到90%。新增转移农村劳动力3万人，实现劳务收入23亿元。农村综合改革进展顺利，农村土地流转面积实现267万亩，增长30.2%。新增农民专业合作社113个。畜牧业规模化、标准化、产业化水平明显提高，牧业产值实现56.3亿元。探索和完善了加快区域发展、推进场县共建的机制和办法，代耕代种代育代管代收“五代”工作和小城镇共建步伐不断加快，合作领域进一步拓宽。圆满完成了第二批新农村建设试点工作，加快了农村城镇化、城乡一体化发展步伐。

——对外开放规模不断扩大。大通道建设取得新进展，新建泊位14个，新增吞吐能力615万吨，全市五个水运口岸的千吨级泊位将达到59个，水运、陆路对俄总运输能力提升到1505万吨。前抚铁路主体工程已完成工程量的90%，同江中俄跨江铁路大桥国内前期工作全部完成；哈佳客运项目国家发改委已立项批复，可研论证完成，即将开工建设；佳木斯国际航空港通过国家验收，并开通韩国首尔的空中航线，在世博会期间开通佳木斯-青岛-上海航线；抚远机场建设取得实质性进展，佳木斯机场迁建、悦来航电枢纽前期扎实推进。鹤大高速公路、哈同高速公路、富绥松花江大桥建设进展顺利。黑瞎子岛生态园基础、主干道路基等工程已完成，上岛桥和防洪工程正在建设。在二十一届哈洽会上签订国外经济技术合作项目10项，金额9561.77万美元，签订国外贸易14.64亿美元，位居全省第三位。成功承办中俄（佳木斯）农机展销洽谈会，达成合作项目总金额7.87亿美元，签订对俄农机及农业类贸易与合作项目3.6亿美元。外经贸主体进一步壮大，进出口实绩企业达到197家，超千万美元的企业53家，超亿美元的企业8家。全市对外贸易进出口总额实现30.5亿美元，增长50%。合作区域进一步拓展，扩大了与俄远东、韩国、意大利等地区和国家的城市友好往来。

——城市建设取得历史性突破。以高水平举办第十二届省运会为牵动，我市城市建设在历史上是投入最多、标准要求最高、建设速度最快、取得成绩最为显著的一年。完成了滨水城市总体规划，推进了“两区、四城、六镇”建设。全力推进了“路桥园林建设百日攻坚战”，新建改造道路38条、桥梁22座，同步实施道路沿线棚户区改造、亮化绿化及各类管线铺设等18项配套工程，工程量是前四年总量的5倍，“一环一带四出口”的城市路网主体框架初步形成，道路交通承载能力大幅度提升。以创建国家园林城为目标，高标准完成了城区内主要建筑548栋、松花江铁路大桥、柳树岛的美化亮化任务，续建新建了双拥、西浦等4个公园。实施春秋两季绿化植树活动，春秋两季万棵杏树和秋季万棵行道树栽种工程取得良好效果。“三个三年”攻坚战全面完成，全年新增供热及并网面积230万平方米，撤消小锅炉56个，政府接管小锅炉18个，春季延长十天停气，秋季提前五天供气，给百姓带来实惠。

新增天然气用户2.6万余户，改造棚户区156.5万平方米，拆迁2.7万余户，市中心区棚户区改造基本完成，2010年是实施棚户区改造以来投资最多和改造最快的一年。土地经营实现新突破，土地出让金实现6.1亿，再创历史新高。城市综合秩序进一步好转，整治脏乱差等“十个专项战役”成效显著，“三废出城”取得明显成效，共清理整治废品收购站181家，“十小”企业362家。关停黑龙农药等“两高一低”企业8家，小造纸企业11家，城区西区日处理10万吨污水处理厂建成试运行。2010年全市好于二级以上良好天数达345天，比2008年增加29天。城市交通秩序的管理水平和市民交通规章制度的遵守有了很大提高。强势整治违章建筑，有效遏制抢建、私建、乱建泛滥势头蔓延，2010年的拆迁量是上年的1.6倍，是拆迁总量最多的一年。强化区级职能，积极探索有效机制，城乡环境卫生状况和冬季清雪状况得到极大改善。14个松花江流域规划治理项目全部完成。

　　——中心城市服务功能全面增强。高新区科技企业孵化器和中俄科技合作中心进展顺利，市区佳南农业科技示范园区和电机股份公司已申报国家级园区和国家级工程中心。我市成为再生资源回收体系建设国家级试点城市。以赫哲文化为核心的敖其湾文化产业园区初具规模并运营，博物馆、图书馆、广电大厦等项目进展顺利；妇联、工会、共青团等人民团体和摄影家协会、工艺美术家协会、红十字会等各类协会发挥积极作用，扩大了城市的知名度。义务教育学校标准化建设不断加强，全部取消所有义务教育公办学校的收费，全市基础教育均衡发展，特别是各县（市）区对基础教育高度重视并取得实质性改观。区域医疗卫生服务中心辐射地位不断巩固，政府投入逐步到位，基层医疗机构药品实行零差价，婚前免费体检工作进一步完善；全民健身活动富有特色，竞技体育取得新突破。我们以举办一届“一流环境、一流设施、一流筹划、一流服务”的盛会为己任，通过3000名快乐舞步队员，5200名演职人员和志愿者的努力，3000多人的警力维护，400余名体育健儿的奋力拼搏，全市上下各单位的无私奉献，第十二届省运会和第八届省少数民族运动会获得圆满成功，向全省人民交上一份满意的答卷，我市在省运会上取得金牌数和团体总分两个第四的历史性突破。成功举办了省第四届残疾人职业技能竞赛。积极探索金融对经济发展支撑服务的有效途径，15家小额贷款公司获准开业，成功争取10亿元城投债券发行，龙江银行佳木斯分行、哈尔滨商业银行富锦支行相继成立，全市八家商业银行2010年共支持全市重点项目、中小企业、城市建设合计贷款62.99亿元；红旗大市场二期、伟嘉千里汽车城等专业市场竣工开业，商贸辐射能力进一步提升，物流业集聚功能进一步增强。高标准建设完成了佳木斯文体会展中心和停建8年的体育场项目，新建改造各类宾馆10家，引进了香格里拉大酒店、中国国旅航空旅游等项目，提升了区域大型文体会展活动接待能力。旅游业发展势头强劲，富锦国家湿地公园、抚远东极等一批重点旅游景区建设加快推进，四大节会影响力不断扩大。

　　——切实保障和改善民生，社会和谐稳定。城乡新就业13.3万人，城镇登记失业率控制在4.43％以下；企业养老保险累计新增参保1.23万人，富锦市和郊区新农合试点新增参保7.4万人，已有60周岁以上农民4.77万人享受到了基础养老金。全市城镇居民医疗参保新增10.03万人，城镇职工基本医疗保险新增参保1.52万人。城市低保户达到12.1万人，农村低保达到12万人，人均补差额达到或超过省定标准。落实廉租实物配租房4886套，新增住房租金补贴1324户。计划生育奖励扶助和独生子女伤残死亡家庭特殊救助政策全面落实，共有4579人得到惠及。扶贫开发第五批整村推进项目进展顺利，低保水平进一步提升。城镇居民人均可支配收入、农民人均纯收入分别达到12186元、7000元，分别增长10.6%、23%。围绕社情民意、源头预防、积案化解和突出问题开展大排查、大化解，排查各类矛盾纠纷742件，调处化解729件，调处化解率98%；排查梳理“三访”案件187件，办结181件，办结率96.8%，稳定率达到90%以上。基层综治维稳工作中心（站、室）实现了全覆盖，人民群众安全感增强。有9个县（市）区被评为省级“平安县（市）区”，桦川县被评为全国“平安县（市）区”。民族宗教工作水平不断提高。安全生产形势进一步好转，连续24年无重大森林火灾，全年无重大安全生产事故发生。

　　——政府自身建设全面加强。坚持依法行政、科学发展、为民执政的理念更加坚定，自觉接受人大依法监督，政协民主监督和社会监督，政府的执行力和公信力显著增强。推行行政问责，取消行政许可和非行政许可226项，对46个部门11类、1600多项行政权力进行规范，清理规范性文件478项，行政效能进一步提升。推行“便捷于民、诚信于法、阳光办公、廉洁高效”服务理念，依法推进公共机构节能，机关服务质量和工作作风不断改善。人大代表建议、政协委员提案办复率100%。

　　各位代表，2010年是“十一五”规划的收官之年。回顾过去的五年，我们深深的感到，在市委“两区两带一城”和十项重点工程战略的引领下，佳木斯已经走出了一条既符合全国、全省发展要求，又符合市情实际的科学发展道路，积累了后发达地区战胜各种困难，解决各种复杂矛盾的宝贵经验，奠定了蓄势待发、加快发展的坚实基础，形成了全市上下万众一心谋发展，想事干事图振兴的良好氛围；过去五年，我们积极应对国际金融危机带来的冲击和挑战，扭住发展不动摇，强化稳定不松劲，实现了地区生产总值、粮食总产、农民人均纯收入五年翻番，固定资产投资和财政总收入两年翻番、工业增加值和财政一般预算收入三年翻番的历史性突破；过去五年，我们牢牢把握规划、建设和管理的每一个环节，着力加强城市基础设施建设，城市承载力显著增强，城市功能不断完善，着力按照“秀气、洋气、灵气、漂亮，幸福指数较高的快乐型城市”标准提档升级，城市知名度和影响力空前提升，城乡面貌发生了根本性变化；过去五年，我们加快体制机制创新，集中力量破解难题，信访积案化解，国企改革全面完成，事业单位、集体林权制度、农村土地流转等重点领域和关键环节改革全面深化，场县共建等区域合作迈出了新步伐；过去五年，我们坚持从群众最期盼的地方做起，从群众最不满意的地方改起，切实保障和改善民生，切实推进平安佳木斯建设，切实改善经济发展环境，社会和谐稳定，人民群众得到更多的实惠。在此，我代表市政府，向全市各族人民，向各民主党派、工商联、各人民团体和各界人士，向驻佳部队、武警官兵致以崇高的敬意！向所有关心、支持佳木斯发展的各界朋友表示衷心的感谢！

　　在充分肯定成绩的同时，我们也清醒的认识到我市经济社会发展中存在的差距和不足：经济发展的质量和效益还有待进一步提高；经济发展结构性矛盾比较突出，二产比重偏小，缺少重大产业项目支撑；非公有制经济和第三产业对经济发展的贡献率还不高；城市基础设施积累矛盾问题还没有有效化解，应急承载能力还不够强，特别是出现冬季供热效果不稳、燃气供应紧张等局面，给群众生活带来不便；社会稳定的任务仍很艰巨，社会保障水平和城市基本公共服务能力还有待于进一步提高；经济发展环境还不够优化，个别部门仍然存在着效率不高、落实不力，不作为或乱作为等问题。这些问题，既是我们更加努力的目标，也是我们工作的出发点，我们一定高度重视，认真解决。

　　二、“十二五”时期经济社会发展的主要任务

　　“十二五”时期是我市发展振兴的重要战略机遇期，也是全面建设小康社会的关键时期，更是深化改革、加快发展方式转变的攻坚时期。市委十一届八次全会对我市经济社会发展面临的形势进行了深入分析，确定了“十二五”时期我市经济社会发展的指导思想、总体思路、目标任务和重大举措。

　　“十二五”时期，我们要以邓小平理论和“三个代表”重要思想为指导，全面贯彻落实科学发展观，坚持发展是硬道理的本质要求，以科学发展为主题，以加快转变经济发展方式为主线，以“两区两带一城”和十项重点工程战略为引领，强化大项目带动、大企业引领、大产业集聚、大园区承载、大市场配套五大支撑。坚定不移推进改革开放，创新科学发展的体制机制；坚定不移推进结构调整，提升产业核心竞争力；坚定不移推进对外开放开发，充分发挥区域优势；坚定不移推进城乡统筹，实现区域及经济社会协调发展；坚定不移推进城市基础设施建设，提升城市的承载能力；坚定不移推进民生改善，保持社会和谐稳定，为全面建成小康社会打下坚实基础。未来五年佳木斯发展一定会进入一个潜力释放期，一定是大有可为的五年，一定是发展更好更快的五年，一定会建设成为经济更加繁荣、社会更加和谐、环境更加优美、人民更加幸福的现代生态宜居城市。

　　“十二五”时期，全市经济社会发展的主要目标是，全市地区生产总值和地方财政收入分别比2010年翻一番。即到2015年，GDP达到1000亿元以上，人均达到37500元。地方财政收入达到50亿元，年均增长20%以上。全社会固定资产投资年均增长30%以上，累计达到2630亿左右。

　　各位代表，站在新的历史起点上，我们要牢牢抓住发展的主动权，时刻保持清醒头脑，认清形势，发挥优势，抢抓机遇，锐意进取，着重把握好以下几点。

一是必须突出加快发展。作为后发达城市，加快发展是我们当前的主要任务。佳木斯的崛起与振兴，保障和改善人民生活都需要加快发展，加快发展是解决一切问题的关键。只有加快发展，才能追赶发达城市，才能补偿历史欠账，才能有效化解各种社会矛盾，才能不断创造更多社会财富，不断为人民谋福祉。同时，加快发展必须是科学的发展，是立足我市实际，充分发挥资源、人才、环境、区位、基础等优势，实现速度、质量和效益相统一的发展。

　　二是必须突出“两区两带一城”和十项重点工程发展战略。实践证明，“两区两带一城”和十项重点工程是市委深入贯彻落实科学发展观的重要成果，是在佳木斯发展实践中探索出的既符合中央和省委的精神，又符合佳木斯实际的科学发展之路；是当前和今后一个时期，引领全市经济社会发展的总体战略。我们要紧紧抓住这个总体战略不动摇、不懈怠、不折腾，一以贯之地抓下去。通过实实在在的项目生成，实实在在的产业发展，获得实实在在的经济总量增长和经济效益的提升。

　　三是必须突出经济结构的战略性调整。我市经济结构矛盾突出，一产和初加工产品比重大，产业链条短，产品附加值低；二产比重小，三产质量和效益不高，特别是缺乏大项目的支撑和大的产业集群。因此，要转变经济发展方式，必须改造提升传统产业，做优存量；必须有产业龙头大项目落地作为牵动，做足增量；必须形成中小企业的规模效益，形成产业集群和聚集效应；必须发展现代服务业，促进就业和提升经济综合实力；必须高度重视金融业在经济社会发展中的作用，增强经济发展的强大动力。

　　四是必须提升城市的承载能力。以打造“三气一亮”的快乐型城市为目标，加大城市基础设施建设。按照“改造老城、建设新区，产业拉动、东拓西优，景区带动、南融北展”总体发展思路，大力实施城市畅通工程，实现立体交通网络格局；大力实施住房保障工程，实现城乡一体化发展的新格局；大力完善基础设施配套工程，实现城镇发展的有效保障；大力提升城市的环境工程，使我市成为全省卫生最干净、秩序最规范、市容最整洁、景观最有特色，环境质量最优的城市。

　　五是必须突出共享改革成果。我市民生历史欠账较多，人民群众要求改善生活环境和质量，提高生活水平的愿望十分迫切。我们必须坚持“万物民为本、万事民为先”的理念，在发展中更加注重社会建设，扩大基本公共服务；更加注重民生工程，促进社会公平正义，让全市人民充分享受改革发展成果，生活更加幸福，更加快乐，更加富有尊严。

　　三、关于2011年主要工作

　　2011年是“十二五”的第一年，也是承前启后的关键之年。巩固“十一五”发展成果，为“十二五”规划起好步，开好局是今年政府工作的主要任务。

今年政府工作的指导思想是：深入贯彻落实中央、省委全会、经济工作会议和市委十一届八次全会精神，以科学发展观为统领，以转变经济发展方式为主线，坚持全市抓发展，重点抓产业，集中力量抓项目，坚持深化改革，扩大开放，改善民生，继续深入实施“两区两带一城”发展战略，全力打好十项重点工程攻坚战。

　　综合考虑各种因素，今年全市主要发展目标是：确保地区生产总值增长13%，规模以上工业增加值增长20%，全社会固定资产投资增长30%以上，财政一般预算收入增长20%，单位GDP综合耗能下降3.5%，化学需氧量排放量下降2%，二氧化碳排放量下降3.5%，城镇人口登记失业率控制在4.5%以内，人口自然增长率控制在5‰以内，城镇居民人均可支配收入增长11%，农民人均纯收入增长11%以上。

按照上述目标，我们将重点推进以下八项工作。

　　（一）大力推进产业项目建设，支撑经济又好又快发展。举全市之力抓项目，深入开展“产业项目攻坚年”活动，加快重大产业项目建设步伐。全年固定资产投资确保实现300 亿元，增长30 %以上，力争超过350亿元，净增近100亿元。重点推进重大项目19项，全年开工建设投资亿元以上重点项目60个，完成产业项目投资120亿元。大力推进工业产业项目。装备制造业通过做大做强和裂变衍生项目，年底达到销售收入80亿元以上。农畜产品加工业推进农业产业化龙头企业建设项目，销售收入达到130亿元以上。新兴产业项目重点推进风电、生物质发电、核电、新材料和新型化工项目，到年底风电总装机容量达到100万千瓦，能源产业销售收入达到35亿元以上，材料和化工产业销售收入达到65亿元以上。区域资源开发利用重点推进和谋划一批利用俄方资源合作开发的重大项目，努力推动产业结构扩展和升级。大力推进以大通道建设为重点的基础设施项目。重点推进哈佳客运专线建设、佳木斯机场迁建立项审批、中俄同江铁路大桥开工、西北绕城高速公路等发展战略项目。大力推进高端服务业项目。推进国际农机具博览中心、佳天农产品交易市场、大世界改造、温州城三期等专业大市场、香格里拉等五星级酒店、大型超市、综合文化服务中心及黑瞎子岛、四丰山、柳树岛综合开发建设。大力推进农田水利项目。重点推进临江、引汤、悦来、幸福、乌苏镇等灌区建设，加快三江灌区、松花江水利航电枢纽申报、开工建设进程，提高农业生产和保障能力。大力推进招商引资工作。各县（市）区和市直重点部门要组织25个以上招商小分队，开展冬季大招商，参加好第五届黑龙江冬季国际投资合作洽谈会等有关活动。全市国内招商引资到位资金实现188.5亿元，增长30%；实际利用外资实现1.3亿元，增长15%。

　　（二）加快结构调整，推动产业结构优化升级和产业聚集。推动经济结构战略性调整，把产业结构调整作为转变发展方式的主攻方向和紧迫任务。一是做大做优工业经济。围绕工业三年再翻番的目标，即到2013年，全市规模以上工业销售收入达600亿元，2011年，全市工业实现销售收入365亿元，增长26%；实现增加值80亿元，增长20%；实现利税30亿元，增长20%。全市工业新增销售收入75亿元。其中，培育新增长点新增销售收入32亿元；骨干企业增产增效新增销售收入35亿元；新进规模企业新增销售收入8亿元。推进企业扩张，加快工业产业成长。全年实施重点工业产业扩张改造52项，投资亿元以上33项，当年完成投资60亿元。推动机械制造、农副产品深加工、新材料及建材、新能源及传统能源、木材造纸、化工制药生物、煤化工等产业全面进步，工业产业实力大幅增强。不断提升企业自主创新能力，争取在重大新产品研发方面取得新突破。二是加快发展以现代服务业为重点的第三产业。以我省东部城市群和对俄贸易为基础，制定现代服务业发展实施规划，开拓各类专业市场，扩大服务种类，提升服务水平和档次，积极培育和发展信息、咨询、会展、创意等新兴服务业，努力扩大城乡及周边城市的需求，开拓城乡市场，推进沃尔玛城市综合体、温馨鸟商务会馆、永安集团商务酒店等项目开工，社会商品零售总额增长15%以上。加强城镇市场监测和调控，进一步开拓农村消费品市场。积极做好“早餐工程试点城市”申报，争取成为全省地市级首批试点城市。大力培育现代物流集团，加快广源物流、天地华宇等重点企业发展，加快佳南浦东货运枢纽站、黑瞎子岛国际货运枢纽等物流园区建设。推进省级和市级8个旅游名镇建设，精心办好四大节会。接待海外旅游者15.8万人次，外汇旅游收入3697万美元，增长10％；接待国内游客277万人次，国内旅游收入11.4亿元人民币，增长15%。三是推进民营经济发展。以建设创业型城市为牵动，大力发展中小企业，制定落实促进中小企业发展的具体措施，建立中小企业专项发展基金，健全发展中小企业考核奖励机制，强化中小企业服务体系建设，完善服务功能，在政策、资金、项目、技术和人才等方面予以重点扶持。四是大力推进园区建设。把园区作为项目落地的载体和带动工业跨越发展的引擎，全力推进园区建设。继续加大力度推进高新技术开发区和各县（市）区工业园区建设。集中建设高新区2平方公里农机产业园的基础设施，完成建设投资10亿元，建成标准厂房10栋。重点推进产业建设项目50个，完成产业项目投资10亿元，确保开工项目30个，高新区雏形基本形成，为创建国家级园区奠定基础。各县（市）区的工业园区要在项目落地同时，加大园区的扩展和基础设施建设，形成园区承接项目的良性发展，年底前园区建设发展要形成一定规模。

　　（三）着力发展现代农业，提高农业综合效益。以建设现代农业综合开发核心区为牵动，全面加快城乡一体化发展。一是提升粮食总产和品质。实施“百亿斤、千万亩”水稻发展规划，新发展水稻100万亩以上。推行规模化生产，对现有1000万亩规模化生产面积提升和规范的同时，各县（市）区继建1-2个10万亩以上的优势种植产业带。实施良种工程，建立水稻、大豆良种繁育基地。二是大幅提高农业综合生产能力。新发展农民专业合作社70个，培育示范典型35个。新建农机合作社100个以上，向省申报投资1000万元以上的农机合作社175个。农机总动力达到300万千瓦，综合机械化程度达到89%。建立健全农业技术标准体系和检测检验体系，种、养标准化水平分别达到80%和60%以上。三是促进农民持续增收。启动“一城带六区”食品加工战略，建设以佳木斯食品加工城为轴心，以六个产业加工园区为支撑，辐射周边地区的经济区格局。引进和培育一批现代化养殖企业，加强重大动物疫病防控，确保畜产品质量安全。牧业产值实现62亿元，增长10%。2011年农业产业化销售收入达到225亿元，增长18.4%。推动农村劳动力向二三产业转移，培训技能农民1万人，转移农村劳动力 35.5万人，创劳务收入23.4亿元。同时，积极培养农民市场意识，为农民搭建产品市场平台，搞好产销对接服务，从农产品终端市场入手，减少中间环节，把利润最大限度留给农民，促进农民增收。四是扎实推进新农村和小城镇建设。各县（市）区要通过典型引路，培育1—2个在基础设施建设、环境建设和产业发展等均衡发展好的试点，提升农村现代化的水平，并不断扩大新农村建设试点面。紧紧抓住与农垦、森工、科研院所的深度合作契机，把小城镇建设作为推进区域城乡一体化发展的重要载体，特别是已经列入“百镇建设工程”的黑瞎子岛、亮子河镇、新城镇等省级试点镇，要确保完成任务。着力抓好寒葱沟镇、鹤立镇等已经列为市级合作共建项目，带动建设一批农业重镇、商贸大镇、边贸强镇、旅游名镇，推进区域一体化进程。

　　（四）进一步扩大沿边开放，增强经济发展活力。充分发挥我市对外开放得天独厚的优势，继续保持外贸稳定增长，对外贸易进出口完成35.05亿美元，增长15%以上。重点支持和培育外贸关联度较强30户出口生产加工企业，扩大出口规模和份额，促进外贸主体企业做大做强。推进口岸建设，由普通口岸向专业口岸转变，推动国际汽车直达客货运输线路延伸、国际集装箱航运开通，并利用江海联运“黄金水道”，进一步向日本、韩国等开放港口拓展。开通佳木斯—哈巴罗夫斯克国际空中航线。积极推进异地签证业务和异地办证边境游。积极开通佳木斯至大连、青岛、三亚等地的航线，加密佳木斯至上海、广州、北京的航线。加强边境管理的有效性，杜绝不利于地区友好的事件发生。加强和俄远东各城市的经贸、科技、文化、教育、体育等友好往来，形成互访机制，促进两个地区间的互补发展。全力推进100万吨电解铝、3万吨海绵钛、鲁能集团犹太自治州煤电一体化等利用俄资源项目建设。高水平举办好第二届中俄农机展，扩大规模，提高效益，总成交额力争达到10亿美元。开工建设我市在俄远东办事处和地方产品展示中心。重点建设黑瞎子岛对俄合作示范区、佳木斯高新区对俄园区、同江桥头经贸合作区和抚远沿边对俄合作示范区四大园区，积极推动同江、抚远、富锦木材加工园区提档升级，形成500万立方米的木材年加工能力。适时推动走出去战略，启动同江在俄下列建立园区的项目。要进一步扩大和港、澳、台的经贸联系，并通过和美国、德国、意大利、韩国等国家的友好城市的建立，扩大交流合作。

　　（五）强化城市承载能力，推进城市建设提档升级。以“七城同创、三优共建”为牵动，实施“城市建设管理升级年”活动，突出抓好规划、建设、配套和综合管理，全面提升城市形象和品位。启动市区生态安全规划设计，城市风貌和滨水景观规划设计，城市市政工程规划设计和城市园林规划设计。以市区松花江第二跨江大桥、悦来航电枢纽、航运新港建设和机场迁建启动为契机，继续按照“以路为纲，配套建设，坚持标准，一步到位”的原则，开工建设城区外环路；打通建设中环路；完善建设内环路，改造建设中环路内的区街巷道。加快推进“两区（四丰山、柳树岛）、四城（滨江、低碳、文化、香格里拉）、六镇（建国、四丰、敖其、长发、江口、望江）”建设，进一步提升城市辐射力和吸引力。组织制定市区供热运行管理规划实施方案，力争国电2台35万千瓦供热机组项目今年开工。完成国电调峰锅炉、华电直排水提热热源系统和达尔凯调峰锅炉完善等热源建设，加快供热管网改造力度，彻底取消供热管网区域内小锅炉。提高供热企业的服务水平，提高政府对供热企业的监管能力。进一步协调天然气气量和用户普及率的关系，探索各种方式提高气源的供给能力，提高天然气应急供气能力。加快城市供水管网区域内自备井的清除力度，实现大网供水，保护水资源，确保饮水卫生安全。实施将城市所有向内河排放的污水全部截流工程，提高建成区内排水管网普及率。深入实施住房保障工程，按照“高端引领，中端扩展，低端保障”的思路，棚户区改造200万平方米，农村泥草房改造8211户。结合文明示范社区建设，启动旧小区综合整治改造；结合物业管理升级，实施楼房保温和平改坡工程。新建廉租住房实物配租3950套。加大园林城建设力度，全面推进“道路、绿带、公园、水系”等绿化建设。深入实施环境质量提升工程，加大对水域、大气、噪音等污染的监控和综合整治，努力改善区域生态环境质量。继续推进“三废”和“十小”出城力度，有效解决居民区污染问题。深入实施市容环境卫生升级工程，推进环卫清雪运行市场化机制，加大建筑残土和装修垃圾的清运整治力度并建立长效机制，彻底解决街路绿带超高土和硬覆盖不足问题。进一步规范整治交通秩序，合理设置停车场和停车位。进一步完善12319服务热线，规范各类经营活动，促进行为遵章有序。进一步严厉打击在城市建设拆迁中突击违建，索要高价影响民心工程的行为，打造更加洁净、有序、优美的城市环境。完善各项工程“规、建、验、管”衔接机制。进一步强化耕地保护制度，大力实施集约和节约用地，不断完善土地利用总体规划。

　　（六）着力推进社会事业建设，促进社会和谐发展。突出抓好群众关心的民生大事。新增城镇就业6.9万人，加大对零就业家庭、残疾人等就业困难人员的就业援助力度。城镇登记失业率控制在4.5%以内。培育城乡创业成功者3000人，拉动就业3万人。抓好城镇个体劳动者、灵活就业人员、农民工、非公有制企业和社会组织人员参保缴费。做好新农保试点的总结验收并扩大试点范围。启动城乡社区示范工程，抓好区域性敬老院和五保家园建设，不断提高集中供养能力。低保补贴发放标准每人每月由221元提高到241元。认真落实好困难家庭、居民和特殊人群的子女上学、住房、供暖、供气、供水等各项救助、补贴政策。继续推进精神病人福利院二期工程、儿救中心等重点社会福利项目的建设。做好第五批93个扶贫开发工作重点村的整村推进工作，实现全市28791名贫困人口脱贫目标。新增1322户廉租住房资金补贴。进一步开展城市“一帮一”扶贫解困、红十字扶危济困等活动，推动慈善事业发展，激发市民仁爱之心，形成仁爱慈善的城市风气。实施学前三年教育三年行动计划，启动公办幼儿园的新建、改扩建工程。推进标准化学校建设，完成市特殊学校改扩建任务。大力发展职业教育，全力支持佳木斯大学博士学位授予单位申办工作，抓好博物馆异地新建项目，做好电台、电视台编播设备试运行。以“快乐舞步”为切入点，推动城乡全民健身工程，探索重振冰雪体育强市有效途径。加大对城市各类文化的挖掘和展示，加快基层公共文化服务体系建设，增建乡镇综合文化站15个以上，全力办好佳木斯全国图书博览会。积极探索医疗改革的有效办法，完善重大疾病防控体系，提高医疗技术水平和服务质量。继续提高机关事业单位公积金缴费比例，由8%提高到10%。加强国防动员、人防工作，切实增强民族宗教工作的主动性和自觉性。重视发展老龄、妇女儿童和残疾人事业，注重人口生育质量。认真落实“米袋子”、“菜篮子”和“油瓶子”行政首长负责制，抓好每个环节，确保米面油和蔬菜食品的有效供给，规范价格调节基金，全市蔬菜种植面积达到56.8万亩。着力强化食品安全和质量的监管，确保城乡居民吃上放心食品，形成食品安全放心的城市形象。树立稳定是第一责任的意识，维护社会安全稳定，认真接待处理群众来信来访，体察民情，妥善解决群众合理诉求，把矛盾化解在基层和萌芽状态。切实建立健全安全生产长效机制，责任到位，监督到位，杜绝重大安全事故发生。强化应急救援管理，做好防灾、抗灾、减灾工作，保证人民群众生命财产安全。深入开展社会治安综合治理，继续以高压态势从速从重从严打击各类犯罪，推进“平安佳木斯”建设。

　　（七）探索破解发展难题，增强经济发展动力。努力破解资金少、土地紧、运输难等发展瓶颈。拓宽融资渠道和方式。通过引导企业规范运营、建立和完善现代企业制度、城镇土地整理、特许经营权经营、资产置换活化等方式扩大与金融机构合作、扩大国有和民营资本的进入，实现经济发展的有效支撑。提高商业银行和政策性银行对企业的服务指导力度，确保完成开行棚户区改造和城市基础设施建设贷款11亿元，确保全市8家商业银行支持重点项目、中小企业，城市建设全年新增贷款100亿元，力争全年投放农业贷款达到120亿元。推动银企合作，防控资金外流，为企业发展提供融资保障。整合政府有效资产，继续开展城投债券融资业务。进一步优化整合市城投公司资产，规范运行，启动上市各项工作。净地出让，经营性用地和工业用地招拍挂率实现100%，土地出让金、年租金、矿产资源补偿费等应收尽收。探索破解运输瓶颈，积极争取国家和省铁路、公路等部门的支持，缓解企业产品运出难问题。认真研究有关政策，争取获得国家更大的建设用地指标支持，为经济发展和大项目落地提供充分的用地保障，重点做好哈佳铁路、高新区及县（市）区工业园区的重大项目征地、供地工作。进一步深化改革，积极探索教、文、体、卫等事业单位改革有效方式；加快县（市）区事业单位改革步伐。继续深化农村综合改革，促进土地流转和规模化经营，积极探索土地股权融资新模式；建立完善林权制度改革配套措施，探索林业经营组织形式。抓住汤原列入国家实施大小兴安岭林区生态保护与经济转型规划的契机，强化森林保护与资源培育。

（八）加强政府自身建设，提高推动科学发展的能力和效率。面对新形势和新任务，我们必须牢记使命，认真履行好人民赋予的权利，心无旁骛，勤奋工作，努力建设勤政、为民、务实、高效、廉洁的政府。一是勇于面对挑战。着力解决发展所面临的各种复杂问题和矛盾，在继续保持良好的精神状态基础上，进一步激发为了发展寝食难安的忘我工作激情；进一步增强不甘人后、奋起直追的志气；进一步坚定不达目的决不罢休的必胜信心。要在加快“两区两带一城”和十项重点工程建设的实践中，敢于触及矛盾，敢于直面问题和困难，解放思想，创造性地解决好各种难题。二是切实转变工作作风。提高行政效能和执政水平，提高政府的执行力、公信力。各级政府工作人员要牢固树立学习意识、责任意识、服务意识，弘扬求真务实的作风，深入实际，掌握实情。坚持讲真话、实话、短话、管用的话，以实实在在的实绩取信于民。要突破传统的思维定势，不断创新工作方式方法，把主要精力放在抓落实上，把心思放在干事业上，把本领用在促进发展上。进一步推行首问责任制、限时办结制、责任追究制等制度，坚决做到失职必纠，渎职必查，维护政令畅通。三是切实推进依法行政。深入贯彻国务院和省政府关于加强法治政府建设文件精神，实施科学、民主、理性的政府决策，使决策更加符合实际，经得起检验。进一步规范行政行为，强化行政问责，坚决纠正行政不作为和乱作为，努力做到规范执法、公正执法、文明执法。自觉接受人大及其常委会的法律监督和工作监督，大力支持政协履行政治协商、民主监督和参政议政职能。认真办好人大代表的议案、建议、批评、意见和政协委员提案。广泛听取民主党派、工商联、无党派人士和各人民团体意见。高度重视司法监督、舆论监督和社会公众监督。四是建设廉洁政府。要认真抓好“一岗双责”党风廉政建设责任制的落实，做到业务工作和廉政建设两手抓、两手硬、两手都能抓好。全力推进政府资源交易平台建设，提高行政审批公开的一站式服务水平，实现政府阳光采购、产权阳光交易，工程阳光招投标，提高政府办公透明度。启动电子政务系统建设，实现政府系统网络办公，文件网上传输和审批。加快市办公中心置换建设，实行集中办公，降低行政成本。进一步加强行政效能监察，严肃查处各类案件。狠抓增收节支，进一步健全非税收入管理机制，推行综合预算，规范行政职能和非税收入收缴管理体制，实行标准公开，统一收费。优化支出结构，集中资金干大事，把有限的资金用到结构调整上，用到人民生活改善上，用到社会事业发展上。

各位代表，“十二五”蓝图已经描绘出佳木斯未来五年的发展美景，实现科学跨越发展，构建和谐佳木斯，是市政府神圣而光荣使命，也是佳木斯人民的共同心愿和热切期盼。让我们在市委的坚强领导下，戮力同心，再接再厉，全面完成今年的各项任务，为谱写佳木斯“十二五”发展的壮丽篇章而努力奋斗！
